# Effect of transcutaneous electrical acupoint stimulation on length of stay for patients after undergoing surgery for colorectal cancer: a systematic review and meta-analysis of randomized controlled trials

**DOI:** 10.3389/fmed.2025.1503714

**Published:** 2025-01-22

**Authors:** YaDan Xiao, Fei Yang, JunLiang Zhou, YuanLing Huang, YaXin Xiao

**Affiliations:** ^1^Binhaiwan Central Hospital of Dongguan, Dongguan, China; ^2^Shenzhen Traditional Chinese Medicine Anorectal Hospital, Shenzhen, China; ^3^The Eighth Affiliated Hospital, Sun Yat-sen University, Shenzhen, China; ^4^Wuhan Institute of Technology, Wuhan, China

**Keywords:** transcutaneous electrical acupoint stimulation, length of stay, colorectal cancer, perioperative management, meta-analysis

## Abstract

**Objective:**

This study aimed to examine the effect of transcutaneous electrical acupoint stimulation (TEAS) on the length of stay (LOS) of patients undergoing surgery for colorectal cancer.

**Methods:**

A systematic review was conducted by searching databases, including PubMed, Embase, Cochrane Library, China Network Knowledge Infrastructure, and Wanfang Database for randomized controlled trials (RCTs) published up to August 24, 2024. Statistical analyses were performed using the Cochrane Collaboration Review Manager (RevMan 5.4). The quality of the RCTs was assessed using the Cochrane Systematic Review Handbook 5.1 and its recommended risk-of-bias assessment tool. Two independent investigators screened and extracted the data and performed statistical analysis.

**Results:**

Seven RCTs were included in the analysis. The findings indicated that TEAS significantly reduced the LOS of patients undergoing surgery for colorectal cancer (mean difference = −1.36, 95% confidence interval = −1.95 to −0.78, *p* < 0.00001). Subgroup analyses of outcome measures, intervention methods, and intervention time points demonstrate the significant effect of TEAS on reducing LOS.

**Conclusion:**

TEAS effectively shortens the LOS of patients undergoing surgery for colorectal cancer. Future studies should focus on refining TEAS protocols and exploring their effects on other aspects of postoperative recovery to fully establish their roles in perioperative management.

## 1 Introduction

Transcutaneous electrical acupuncture stimulation (TEAS) is an innovative, noninvasive technique that involves the application of electrical stimulation to specific acupoints on the skin to replicate the effects of traditional electroacupuncture without the use of needles (1). Compared with pharmacological interventions, TEAS has gained attention in the perioperative period because of its noninvasive nature and minimal side effects (2). Its role in promoting postoperative recovery is rapidly being recognized, particularly in pain control (3), gastrointestinal function improvement (4), and reduction of inflammation (5).

Colorectal cancer surgery, a major surgical intervention, is often accompanied by significant postoperative challenges, including postoperative ileus, pain, and delayed gastrointestinal recovery, leading to prolonged hospital stays (6). The length of stay (LOS) is a key indicator for assessing postoperative recovery (7), with prolonged LOS associated with increased risks of hospital-acquired infections, higher medical costs, and reduced patient satisfaction. Given the economic burden and clinical implications, exploring strategies to effectively reduce LOS and improve recovery time for patients with colorectal cancer is essential.

Although TEAS has been evaluated for its effects on postoperative recovery in various surgical contexts, its specific effect on LOS in patients with colorectal cancer remains underexplored. Thus, this meta-analysis aimed to systematically assess the effectiveness of TEAS in reducing LOS among patients undergoing surgery for colorectal cancer, thereby providing insights into its potential as a beneficial adjunct therapy.

## 2 Materials and methods

### 2.1 Study design and protocol

This meta-analysis was conducted following the Preferred Reporting Items for Systematic Reviews and Meta-Analyses guidelines (8). This study involved a literature review and meta-analysis, so the need for ethical approval or informed consent was waived.

### 2.2 Search strategy

A comprehensive literature search was performed across multiple databases including PubMed, Embase, Cochrane Library, China Network Knowledge Infrastructure, and Wanfang Database up to August 24, 2024. The following keywords were used in the search: (((((Length of Stay) OR (Hospital stay)) OR (Length of hospitalization)) OR (hospitalization time)) AND (((colorectal cancer) OR (colon)) OR (rectal))) AND ((((transcutaneous electrical acupoint stimulation) OR (transcutaneous acupoint electrical stimulation)) OR (TEAS)) OR (TAES)). The search strategy combined Medical Subject Headings and free text terms to capture all relevant studies. The reference lists of selected studies were also reviewed to identify additional studies.

### 2.3 Inclusion criteria

Eligible studies were prospective randomized controlled trials (RCTs). We defined our inclusion criteria based on the PICO framework: (P) Population: patients with colorectal cancer who underwent surgery. (I) Intervention: TEAS. (C) Control: sham TEAS or non. (O) Outcome: LOS. Studies must provide quantifiable data on the LOS. No restrictions were placed on the age, sex, or nationality of the participants.

### 2.4 Exclusion criteria

Studies were excluded if they were reviews, case reports, or non-RCTs or if they did not provide measurable LOS-related outcomes. Studies focusing on other forms of acupuncture or non-acupuncture therapies were also excluded.

### 2.5 Article screening and data extraction

Two independent reviewers screened the titles and abstracts for relevance and obtained the full texts of potentially eligible studies. Data extraction was independently conducted by two reviewers using a standardized data extraction form to gather pertinent information. The extracted data included details such as the name of the primary author, year of publication, baseline characteristics of the participants (including population size and age), procedural specifics of the treatment and control groups, outcome indicators, and other relevant literature data. Discrepancies were resolved through discussion or consultation with a third reviewer.

### 2.6 Quality assessment

Adhering to the Cochrane Systematic Review Handbook 5.1 and its recommended risk-of-bias (RoB 2.0) assessment methodology, the quality of the included studies was thoroughly examined. This evaluation encompassed scrutiny of the following factors: sequence generation (selection bias), allocation concealment (selection bias), blinding of participants and personnel (performance bias), blinding of outcome assessment (detection bias), incomplete outcome data (attrition bias), selective reporting (reporting bias), and other biases. The results of the risk-of-bias assessment were categorized into “low risk,” “high risk,” and “unclear.” Also, assess the quality of the studies using the Grading of Recommendations Assessment, Development, and Evaluation (GRADE) methodology.

This quality evaluation was conducted independently by two researchers, and any conflicts were resolved through mediation by the corresponding author.

### 2.7 Statistical analysis

The meta-analysis was conducted using Cochrane Collaboration Review Manager (RevMan 5.4). Continuous data are expressed as mean difference (MD) with 95% confidence intervals (CIs), whereas dichotomous data are presented using relative risk with 95% CIs. Results from all studies were pooled to assess differences in the efficacy of the interventions. Subgroup analyses were conducted based on different TEAS protocols and patient characteristics.

The statistical heterogeneity between studies was evaluated using the I^2^ statistic, where I^2^ values of ≤50 and > 50% indicated the absence and presence of substantial heterogeneity, respectively. We used random effects models with 95% confidence intervals for the analysis.

## 3 Results

### 3.1 Search and study selection

**Figure 1:**
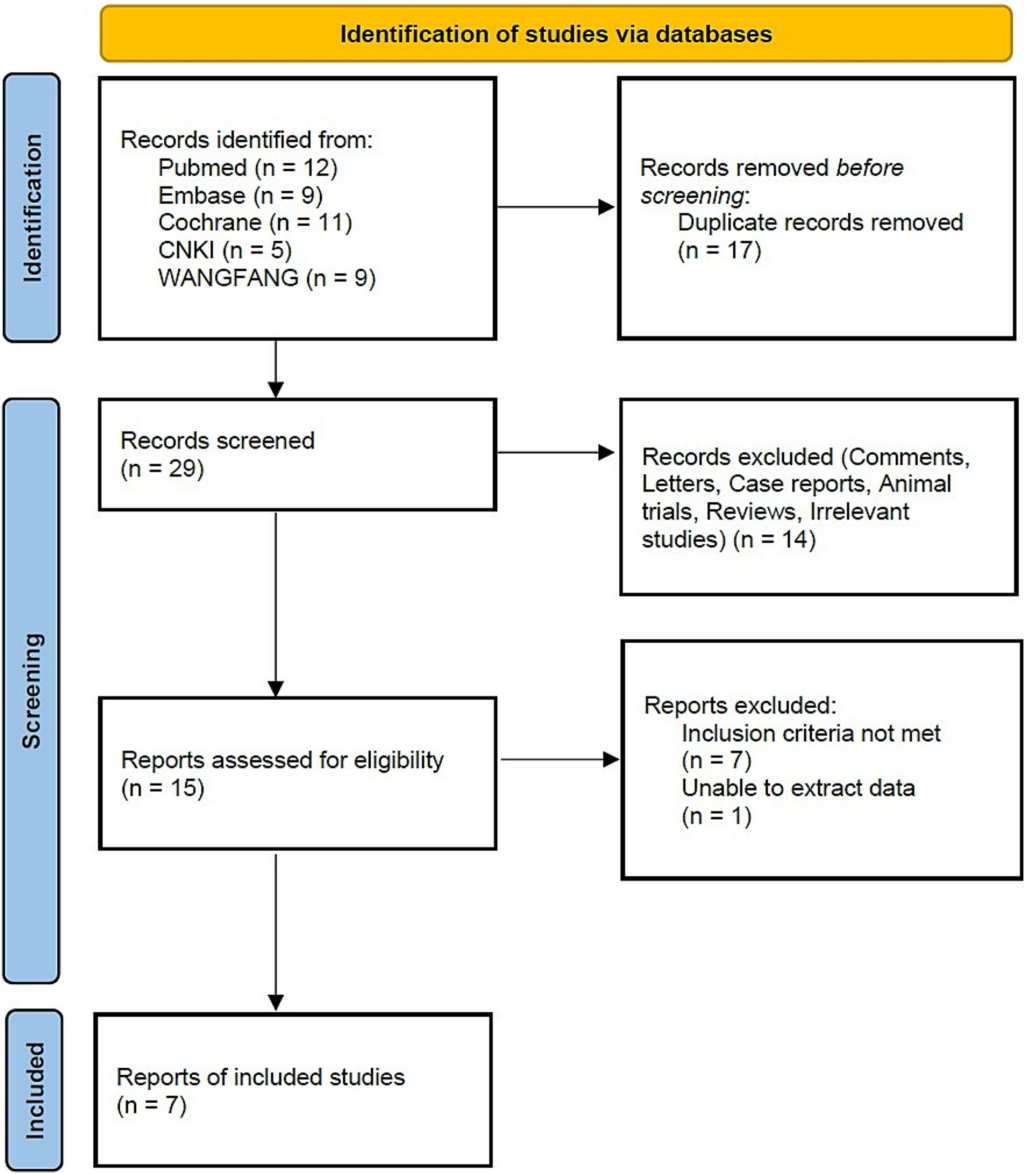
Flow diagram.

A total of 46 studies were identified from the initial search. After removing duplicates, 29 studies were screened. Following the screening process, 15 studies were assessed based on their full text, and 7 studies (9–15) met the inclusion criteria for this meta-analysis. The detailed process is illustrated in Figure 1.

### 3.2 Study characteristics

The included studies were published between 2017 and 2023, involving a total of 562 patients with colorectal cancer (TEAS group, *n* = 282; control group, *n* = 280). These studies varied in terms of TEAS protocols, including differences in stimulation time points, acupoints, and frequency. The specific characteristics of the included studies are presented in Table 1.

**Table 1:** Characteristics of the included trials.

Study ID	TEAS group			Control group			Result	Acupoint	Stimulus timing	GRADE
	Sample size	Age	Intervention	Sample size	Age	Intervention				
Yuan et al., 2017 (9)	30	53.9 ± 9.8	TEAS	30	54.6 ± 10.4	Sham-TEAS	LOS	Neiguan (PC6), Hegu (LI4), Zusanli (ST36)	Pr, In, Po	High
Fan et al., 2018 (10)	26	54 ± 7	TEAS	26	54 ± 8	Non	PLOS	Neiguan (PC6), Hegu (LI4), Zusanli (ST36), Shangjuxu (SP6), Xiajuxu (ST39)	Pr, In	Moderate
Wei et al., 2019 (11)	52	60.21 ± 9.74	TEAS	52	60.22 ± 9.83	Non	LOS	Hegu (LI4), Quchi (LI11), Neiting (ST44), Zusanli (ST36)	Po	High
Huang et al., 2019 (12)	29	58.59 ± 11.27	TEAS	28	60.57 ± 12.1	Sham-TEAS	PLOS	Zusanli (ST36)	Pr, In	High
Liu et al., 2021 (13)	50	70.8 ± 5.41	TEAS	50	69.68 ± 4.85	Sham-TEAS	LOS	Neiguan (PC6), Hegu (LI4), Zusanli (ST36)	Pr, In	Moderate
Lu et al., 2022 (14)	47	56.2 ± 9.0	TEAS	47	55.6 ± 9.9	Sham-TEAS	LOS	Neiguan (PC6), Zusanli (ST36)	Pr, Po	High
Li et al., 2023 (15)	48	58.12 ± 7.34	TEAS	47	56.45 ± 7.26	Sham-TEAS	PLOS	Neiguan (PC6), Hegu (LI4), Zusanli (ST36), Sanyinjiao (SP6)	Pr, In	Moderate

TEAS, transcutaneous electrical acupoint stimulation; LOS, length of stay; PLOS, postoperative length of stay. In, intraoperative; Po, postoperative; Pr, preoperative.

### 3.3 Risk-of-bias and study quality assessment

**Figure 2:**
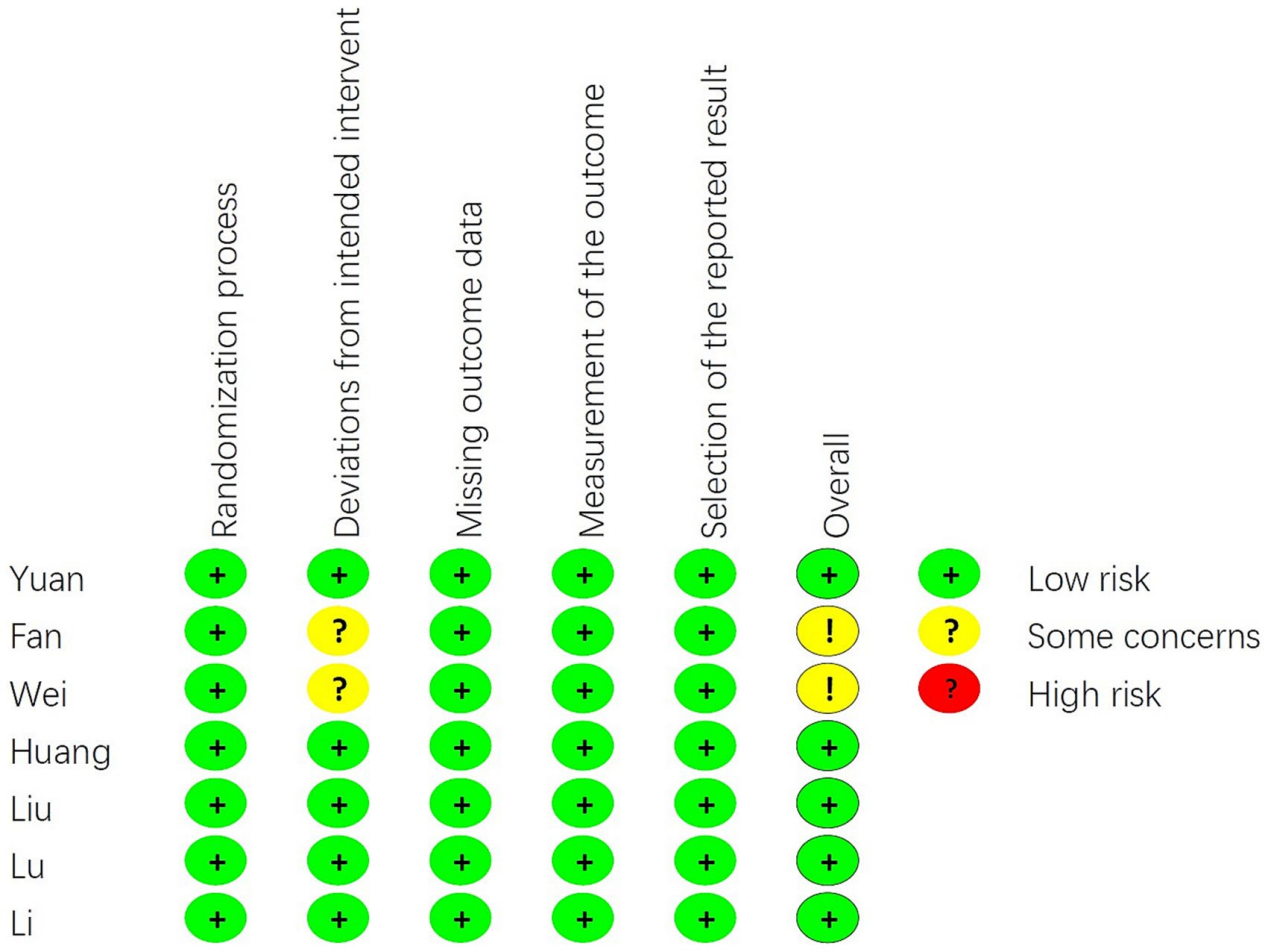
The risk of bias graph in the single study.

**Figure 3:**
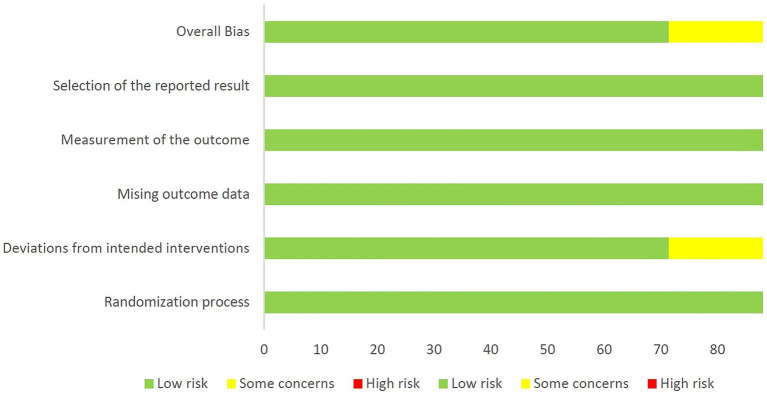
The risk of bias graph in the average of all included studies.

After conducting a quality assessment using the Cochrane Risk-of-Bias Assessment Tool on the seven included publications, the overall quality of the entire literature was evaluated as good (Figures 2, 3). As for study quality assessment, 4 (57.1%) studies were rated as high and 3 (42.9%) were rated as moderate (Table 1).

### 3.4 Synthesis of results

**Figure 4:**
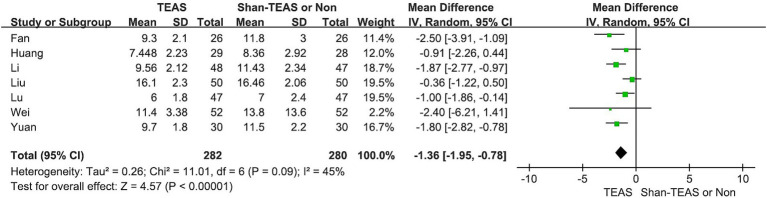
Effect of TEAS on the length of stay.

The meta-analysis of the seven studies revealed a significant reduction in the LOS in the TEAS group compared with the control group (MD, −1.36; 95% CI, −1.95 to −0.78; *p* < 0.00001; Figure 4).

**Figure 5:**
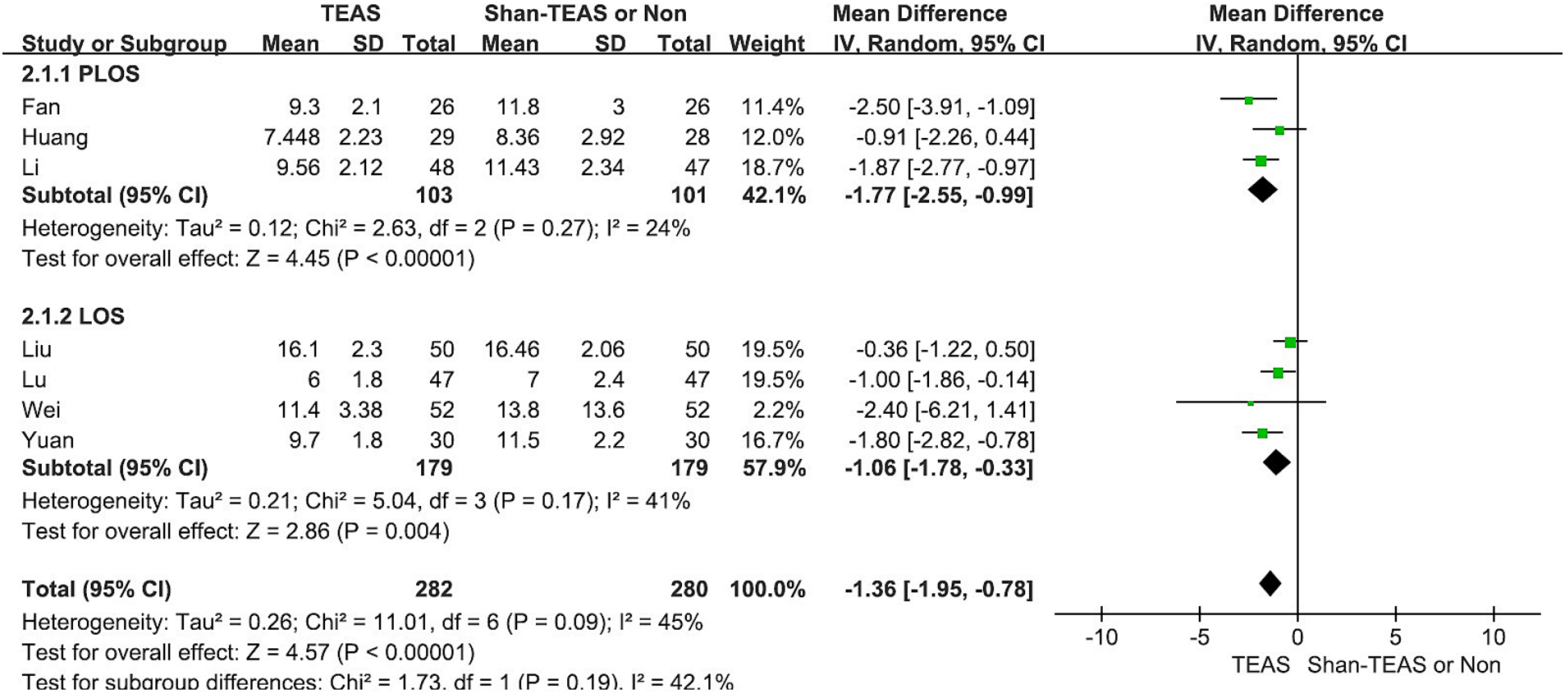
Forest plot of the subgroup analysis of the effect of TEAS on length of stay and postoperative length of stay.

In the first subgroup analysis, TEAS was found to reduce both the LOS and postoperative LOS (PLOS) compared with the control intervention (MD, −1.06; 95% CI, −1.78 to −0.33; *p* = 0.004; MD, −1.77; 95% CI, −2.55 to −0.99; *p* < 0.00001; Figure 5).

**Figure 6:**
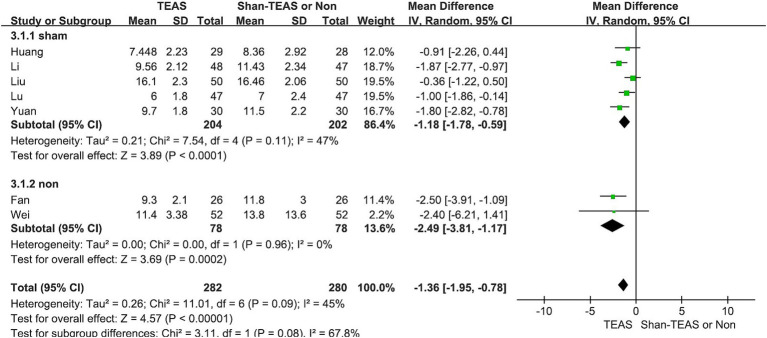
Forest plot of the subgroup analysis of the effect of TEAS compared with sham TEAS and control without intervention.

In the second subgroup analysis, the TEAS treatment revealed significant differences compared with the sham TEAS or control groups (MD, −1.18; 95% CI, −1.78 to −0.59; *p* < 0.00001; MD, −2.49; 95% CI, −3.81 to −1.17; *p* = 0.0002; Figure 6).

**Figure 7:**
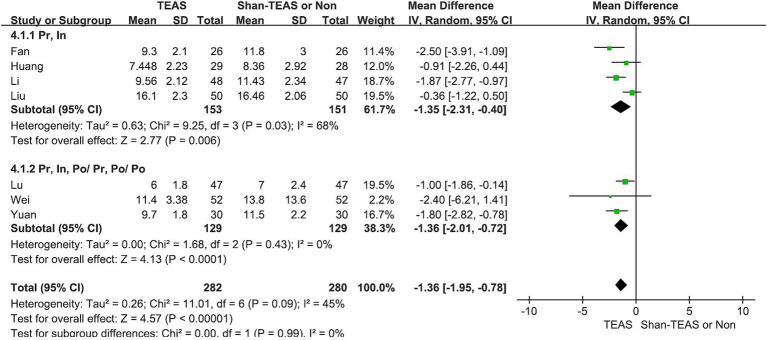
Forest plot of the subgroup analysis of the effect of TEAS at different time points.

In the third subgroup analysis, TEAS was found to reduce the LOS at both preoperative and intraoperative intervention time points compared with other treatment time points (MD, −1.35; 95% CI, −2.31 to −0.40; *p* = 0.006; MD, −1.36; 95% CI, −2.01 to −0.72; *p* < 0.0001; Figure 7 and Table 2).

**Table 2:** Subgroup analysis.

Subgroup	No of study	Sample size	Heterogeneity I^2^	MD	*p*
Results					
LOS	4	358	41%	−1.06(−1.78, −0.33)	0.004
PLOS	3	204	24%	−1.77(−2.55, −0.99)	<0.00001
Intervention comparison					
Sham-TEAS	5	406	47%	−1.18(−1.78, −0.59)	<0.00001
Non	2	156	0%	−2.49(−3.81, −1.17)	0.0002
Stimulus timing					
Pr, In	4	304	68%	−1.35(−2.31, −0.40)	0.006
Pr, In, Po/ Pr, Po/ Po	3	258	0%	−1.36(−2.01, −0.72)	<0.0001

In, intraoperative; Po, postoperative; Pr, preoperative.

### 3.5 Publication bias and sensitivity analysis

**Figure 8:**
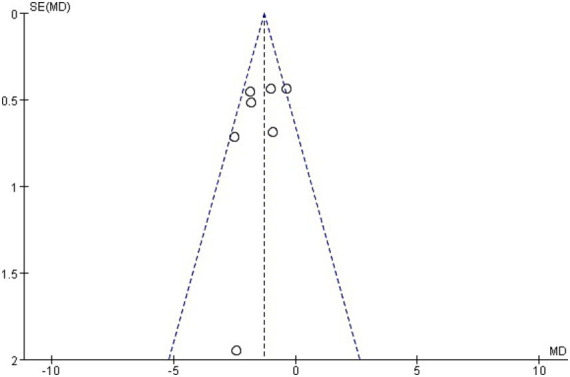
Funnel plot of the length of stay.

The Funnel plot suggested that there was no evidence of publication bias in studies discussing the effects of TEAS on LOS (Figure 8). Then, sensitivity analysis was performed to evaluate the stability of the result, resulting in the omission of one study from the meta-analysis at a time. The result revealed no significant change in the corresponding merged estimate (Table 3).

**Table 3:** Sensitivity analysis results after removing one study at a time.

Removed study	MD	95% CI	P_heterogeneity_	I^2^
Fan (2018)	−1.21	−1.77, −0.65	<0.0001	37%
Huang (2019)	−1.44	−2.11, −0.77	<0.0001	53%
Li (2023)	−1.25	−1.91, −0.59	0.0002	45%
Liu (2021)	−1.57	−2.04, −1.10	<0.00001	2%
Lu (2022)	−1.47	−2.18, −0.75	<0.0001	52%
Wei (2019)	−1.34	−1.96, −0.73	<0.0001	53%
Yuan (2017)	−1.28	−1.96, −0.61	0.0002	49%

## 4 Discussion

The results of this meta-analysis indicate that TEAS significantly reduces LOS among patients undergoing colorectal cancer surgery (MD, −1.36; 95% CI, −1.95 to −0.78; *p* < 0.00001). Subgroup analyses based on outcome measures (MD, −1.06; 95% CI, −1.78 to −0.33; *p* = 0.004; MD, −1.77; 95% CI, −2.55 to −0.99; *p* < 0.00001), intervention methods (MD, −1.18; 95% CI, −1.78 to −0.59; *p* < 0.00001; MD, −2.49; 95% CI, −3.81 to −1.17; *p* = 0.0002), and intervention time points (MD, −1.35; 95% CI, −2.31 to −0.40; *p* = 0.006; MD, −1.36; 95% CI, −2.01 to −0.72; *p* < 0.0001) consistently demonstrated the significant effects of TEAS on reducing the LOS, highlighting its potential as an effective perioperative intervention.

All seven studies included in the analysis involved laparoscopic surgery. Compared with traditional open surgery, laparoscopic procedures significantly shorten postoperative LOS and reduce complication rates (16). However, laparoscopic surgery can lead to anatomical abnormalities and impaired bowel function caused by bowel tissue removal, as well as mucosal ischemia and hypoxia from the pneumoperitoneum, which may further disrupt gastrointestinal function. Therefore, perioperative gastrointestinal recovery remains a clinical challenge that is crucial for reducing the LOS.

The reduction in the LOS associated with TEAS can be attributed to several mechanisms. First, TEAS has been shown to improve bowel function (17) and reduce the incidence of postoperative bowel obstruction (18), thereby promoting gastrointestinal recovery. TEAS may regulate the brain–gut axis, thereby enhancing gastrointestinal motility and accelerating the return to normal bowel function (4). In addition, TEAS can reduce the LOS by alleviating postoperative pain and decreasing the need for analgesics (19). The analgesic effect of TEAS is thought to be mediated by the release of endogenous opioids, such as *β*-endorphins, which modulate pain perception and reduce opioid consumption (20). The use of TEAS during the perioperative period increased the serum β-endorphin levels of the patients (21). The reduction in pain and opioid use not only enhances patient comfort and may accelerate recovery and reduce LOS.

However, variability in TEAS protocols, such as differences in the stimulation frequency, intensity, and duration, may contribute to the heterogeneity observed in the results. Among these factors, the timing of the intervention (preoperative, intraoperative, or postoperative) may influence the time to the first postoperative gas and bowel movement. Our subgroup analysis shows that the results remain comparable across different time points. This provides a basis for selecting the optimal clinical intervention timing and suggests that continuous TEAS treatment may not be necessary. Future studies should focus on standardizing TEAS protocols to determine the most effective parameters and maximize patient benefits. Although the current analysis indicates a significant reduction in the LOS, the clinical effect of TEAS on other important outcomes, such as postoperative complications and overall recovery time, warrants further investigation.

Incorporating TEAS into standard postoperative recovery protocols could significantly affect healthcare systems by reducing hospital costs and improving patient outcomes. Previous studies have highlighted similar benefits for other surgical populations (5, 18, 22), suggesting that TEAS could be a valuable for managing postoperative recovery across various surgical fields. Through the data analysis of 562 participants, along with sensitivity and heterogeneity analyses of the results, we believe that the conclusion is reliable. However, the quality of evidence and limitations in the study design, such as the small sample size and variability in study quality, highlight the need for further high-quality, large-scale trials to validate these findings and optimize TEAS protocols. The limitations, such as inconsistent intervention protocols, should be taken into account when interpreting the findings of this study. Further well-designed trials that address these limitations are needed to provide more robust and reliable evidence.

In summary, TEAS is a promising approach for reducing the length of stay (LOS) in patients undergoing surgery for colorectal cancer, with demonstrated clinical feasibility and operational applicability. It plays an important role in Enhanced Recovery After Surgery protocols, offering potential benefits in improving clinical outcomes and reducing healthcare costs. Future research should focus on standardizing TEAS protocols and conducting large-scale, multicenter randomized controlled trials.

## Data Availability

The original contributions presented in the study are included in the article/supplementary material, further inquiries can be directed to the corresponding author.
